# Vitamin D Supplementation and Breast Cancer Prevention: A Systematic Review and Meta-Analysis of Randomized Clinical Trials

**DOI:** 10.1371/journal.pone.0069269

**Published:** 2013-07-22

**Authors:** Francesca Sperati, Patrizia Vici, Marcello Maugeri-Saccà, Saverio Stranges, Nancy Santesso, Luciano Mariani, Antonio Giordano, Domenico Sergi, Laura Pizzuti, Luigi Di Lauro, Maurizio Montella, Anna Crispo, Marcella Mottolese, Maddalena Barba

**Affiliations:** 1 Bostatistics/Scientific Direction, Regina Elena National Cancer Institute, Rome, Italy; 2 Division of Medical Oncology B, Regina Elena National Cancer Institute, Rome, Italy; 3 Division of Medical Oncology B/Scientific Direction, Regina Elena National Cancer Institute, Rome, Italy; 4 Health Sciences, Warwick Medical School, Coventry, United Kingdom; 5 Department of Clinical Epidemiology and Biostatistics, McMaster University Health Sciences Centre, Ontario, Canada; 6 Division of Gynecologic Oncology, Regina Elena National Cancer Institute, Rome, Italy; 7 Sbarro Institute for Cancer Research and Molecular Medicine and Center of Biotechnology, Temple University, Philadelphia, Pennsylvania, United States of America; 8 Epidemiology Unit, National Cancer Institute G. Pascale Foundation, Naples, Italy; 9 Department of Pathology, Regina Elena National Cancer Institute, Rome, Italy; Universidad Peruana Cayetano Heredia, Peru

## Abstract

In recent years, the scientific evidence linking vitamin D status or supplementation to breast cancer has grown notably. To investigate the role of vitamin D supplementation on breast cancer incidence, we conducted a systematic review and meta-analysis of randomized controlled trials comparing vitamin D with placebo or no treatment. We used OVID to search MEDLINE (R), EMBASE and CENTRAL until April 2012. We screened the reference lists of included studies and used the “Related Article” feature in PubMed to identify additional articles. No language restrictions were applied. Two reviewers independently extracted data on methodological quality, participants, intervention, comparison and outcomes. Risk Ratios and 95% Confident Intervals for breast cancer were pooled using a random-effects model. Heterogeneity was assessed using the I^2^ test. In sensitivity analysis, we assessed the impact of vitamin D dosage and mode of administration on treatment effects. Only two randomized controlled trials fulfilled the pre-set inclusion criteria. The pooled analysis included 5372 postmenopausal women. Overall, Risk Ratios and 95% Confident Intervals were 1.11 and 0.74–1.68. We found no evidence of heterogeneity. Neither vitamin D dosage nor mode of administration significantly affected breast cancer risk. However, treatment efficacy was somewhat greater when vitamin D was administered at the highest dosage and in combination with calcium (Risk Ratio 0.58, 95% Confident Interval 0.23–1.47 and Risk Ratio 0.93, 95% Confident Interval 0.54–1.60, respectively). In conclusions, vitamin D use seems not to be associated with a reduced risk of breast cancer development in postmenopausal women. However, the available evidence is still limited and inadequate to draw firm conclusions. Study protocol code: FARM8L2B5L.

## Introduction

In recent years, the scientific evidence linking vitamin D (vit D) to breast cancer has grown notably. Garland and Garland first hypothesized a role of exposure to solar radiation in explaining geographic variation in breast cancer incidence. Accordingly, lower levels of vit D resulting from weaker UV-B radiation were suggested to explain higher breast cancer rates at higher latitudes. However, this ecological observation was only partially substantiated by subsequent epidemiological studies [1]–[11].

Several observational studies have focused on the association between breast cancer risk and circulating levels of 25 (OH) hydroxyvitamin D (25-OH vit D), which is the precursor of the active hormone 1,25 (OH)2 vit D and the most commonly used biomarker of vit D status. Results from case-control studies have consistently revealed an inverse association between 25-OH vit D and breast cancer [12]–[14]. Conversely, evidence from prospective studies tend to be inconsistent. No significant inverse association between 25-OH vit D levels and breast cancer risk was observed in a meta-analysis including four prospective studies in 2010 [12], while in a subsequent meta-analysis including two additional prospective studies a significant inverse association was found [13]. Since negative findings emerged from three further prospective studies published after these latter meta-analyses [15]–[17], the evidence from prospective studies focused on the association between 25-OH vit D levels and breast cancer risk remains substantially unclear.

Several systematic reviews including randomized controlled trials (RCTs) have recently focused on vit D and health outcomes. Autier investigated the impact of vit D supplementation on death from any cause including cancer. Vit D was associated with a slight reduction in death from any cause [summary relative risk and 95% Confident Interval (CI) were 0.93, 0.87–0.99]. Eighteen RCTs were included, but only two of them reported on cancer incidence and mortality, overall and for colorectal cancer [18]–[20]. Chung has addressed the role of vit D supplementation in prevention of cancer and fractures. Nineteen RCTs were included, but only three focused on cancer outcomes and two reported on breast cancer [21]–[23]. Though limited, the available data seemed to suggest a role of vit D in reducing the risk for total cancer [23]. More recently, an individual patient data meta-analysis of eight RCTs has confirmed a 7% reduction in overall mortality for participants allocated to vit D (0.93, 95% CI 0.88–0.99). The authors did not report on cancer outcomes [24].

So far, no systematic review has specifically addressed the role of vit D supplementation in breast cancer prevention. We aimed to investigate risk of breast cancer development in a systematic review of women participating in RCTs of vit D supplementation compared with placebo/no treatment.

## Materials and Methods

This systematic review was performed in full agreement with an ad-hoc study protocol which was submitted to the Italian Agency of Drugs (AIFA) in 2008 (study protocol code: FARM8L2B5L).

### Data Sources and Search

In April 2012, a qualified librarian used OVID to electronically search MEDLINE (R) (January 1950 onward), EMBASE (January 1980 onward), and the Cochrane Central Register of Controlled Trials (CENTRAL) (The Cochrane Library, latest issue). We designed and applied the search strategy using sensitivity criteria potentially capturing RCTs of vit D use in both breast cancer prevention and treatment. To this aim, we combined terms for vit D and cancer (Appendix 1) with search filters for RCTs [25]. We also screened the references of included studies and used the “Related Article” feature in PubMed to identify additional articles.

No language restrictions were applied.

### Study Selection and Outcomes of Interest

Included studies were RCTs of vit D in breast cancer. Studies suitable for inclusion were RCTs of vit D in breast cancer. We considered RCTs if vit D was administered as a single agent compared with placebo/no treatment or as part of combined regimens including supplements and lifestyle modifications as long as the administration of the co-intervention was planned to be the same in all groups. For multi-arm RCTs, we included all pairwise comparisons with arms differing by vit D use only. Breast cancer incidence and mortality were the outcomes of interest in RCTs focused on breast cancer prevention. We planned to consider survival, time to recurrence, risk of ipsilateral and/or contralateral disease, health related quality of life and toxicity as outcomes of interest in RCTs of vit D in breast cancer treatment. However, only trials of vit D in breast cancer prevention were included. RCTs involving pregnant or lactating women were excluded.

### Data Extraction and Assessment of Risk of Bias

Two reviewers independently completed title and abstract screening and full text review. Disagreements were solved through discussion or consultation of a third reviewer. A pilot-tested form was used for data extraction. Collected data related to methodological quality, participants, intervention, comparison and outcomes. In regards to outcome data, Risk Ratios (RRs) were calculated by treatment arm for breast cancer incidence. For mortality, we planned to abstract the log hazard ratio [log(HR)] and its variance [26].

Risk of bias was assessed using the Cochrane risk of bias tool. Two reviewers independently assessed methodological quality and resolved disagreements by discussion. Their evaluation focused on randomization, blinding, percentage of lost to follow-up, early stop for benefit or harm, intention-to-treat (ITT) principle, incomplete outcome data, selective reporting. In order to assess reporting bias, we compared the list of outcomes from the protocol to the outcomes reported in the published paper [27]. We planned to assess publication bias by mean of funnel plots and visual inspection for asymmetry [28]–[29]. However, the very low number of RCTs included did not allow such an evaluation.

### Data Synthesis and Analysis

We calculated the agreement between the two reviewers for the assessment of eligibility using kappa statistic.

The RRs for breast cancer development were pooled using the Der Simonian-Laird random-effects model [29]. Data on breast cancer mortality were reported only in one single study [30].

Heterogeneity was assessed using the I^2^ test and judged considerable if ≥75% [29]. We tested the effect of vit D dosage in meta-regression analysis. In subgroup analyses, we assessed whether vit D administration as a single agent or combined with calcium had an impact on treatment effects. We used Revman 5.0 and Stata version 8.2 (Stata Coro., College Station, Tx, USA) for statistical analysis.

## Results

### Results of the Search

**Figure 1:**
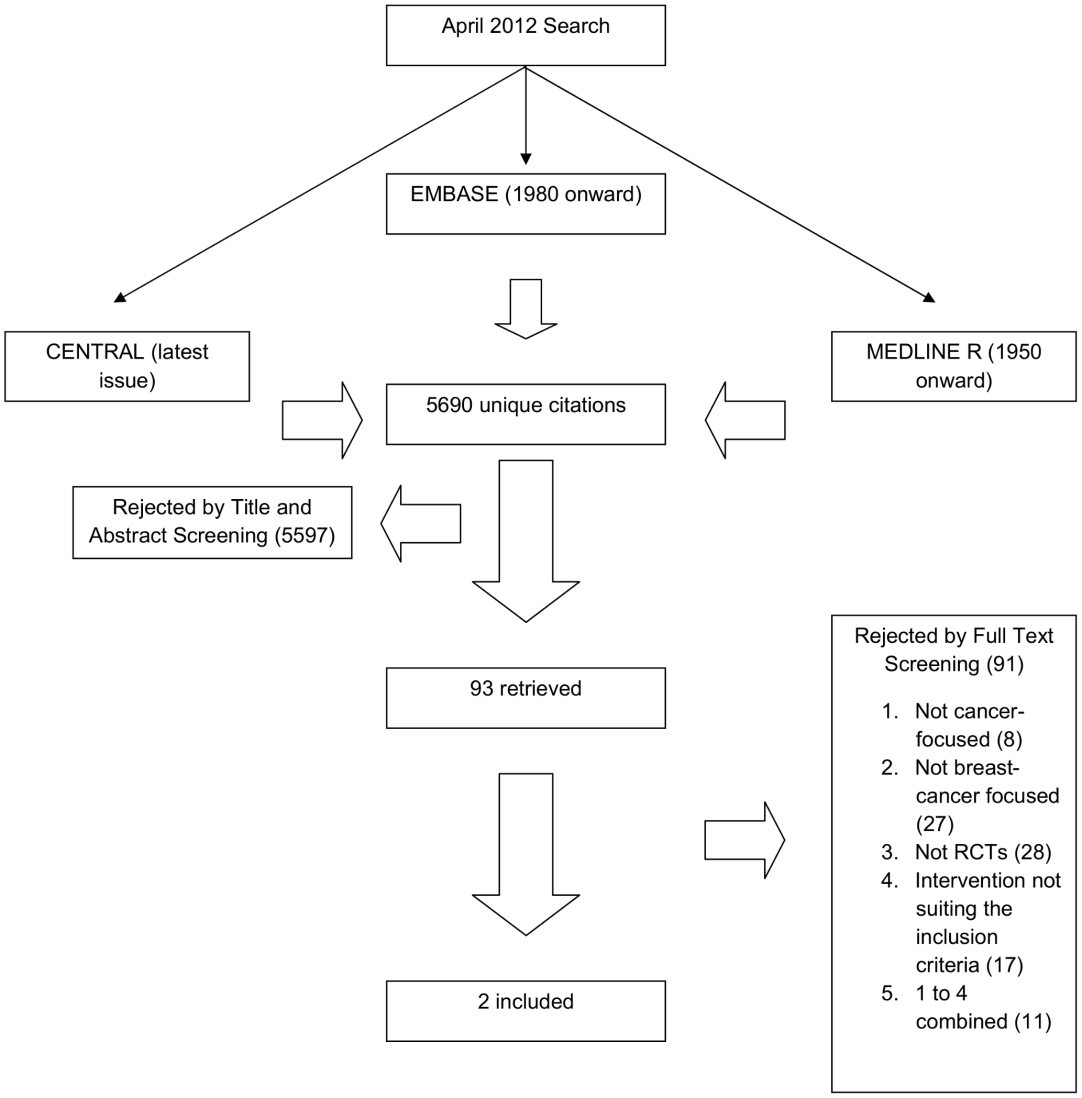
Flow diagram of the trial selection process.

The flow diagram of the trial selection process appears in figure 1.

The April 2012 search identified 5690 unique citations. Based on the title and abstract screening, we rejected 5597 citations. Among the remaining 93 unique citations, 91 were excluded for the following reasons: 1. Eight did not report on cancer outcomes [31]-[38]; 2. Twenty four did not report on breast cancer outcomes [39]–[62]; 3.Twenty eight were not RCTs [63]–[90]; 4. Eighteen did not fulfill the inclusion criterion related to the intervention [22], [91]–[107]; 5. Thirteen were excluded because combining at least 2 among the previously described features (1 to 5) [18], [20], [108]–[118] (Figure 1). Only two trials fulfilled the inclusion criteria [21], [30]. Agreement between reviewers for study eligibility was excellent (kappa = 0.99).

### Included Studies

Table 1 lists the characteristics of the two studies included.

**Table 1:** Characteristics of the included Randomized Clinical Trials (RCTs).

RCTs	Characteristics	Description
Avenell A, 2012 [30]	Methods	Randomized, placebo-controlled trial. Participants were recruited from fracture clinics or orthopedic wards. Randomization was computer generated, stratified by center, and minimized by age, gender, time since fracture. Allocation Concealment: centralized randomization. ITT analysis: yes. Blinding: not clear. Incomplete outcome data: not clear. Stopped early for benefit/arm: no.
	Participants	5292 people aged at least 70 years with previous low-trauma fracture. Calcium plus vitamin D: 1306 patients; F/M: 1104/202; mean age: 78±6; Breast cancers: 20/1306. Calcium: 1311 patients; F/M: 1113/198; mean age: 77±6; Breast cancers: 21/1311. vitamin D: 1343 patients; F/M: 1136/207; mean age: 77±6; Breast cancers: 23/1343. Placebo: 1332 patients; F/M: 1128/204; mean age: 77±6; Breast cancers: 16/1332.
	Interventions	Participants were randomized into four equal groups to receive two tablets daily containing a total of 800 IU vitamin D, 100 mg elemental calcium, both vitamin D and calcium, or placebo for 24–62 months, with a minimum follow-up of 3years after intervention.
	Outcomes	All-cause mortality, mortality due to vascular disease and cancer, cancer incidence.
Lappe JM, 2007 [21]	Methods	Population-based, double-blind, randomized placebo-controlled trial. Participants were recruited as a population-based sample from a 9-county, largely, rural area in Eastern Nebraska, with the use of random telephone dialing of all listed telephones in the counties concerned. Allocation Concealment: centralized randomization. ITT analysis: yes. Blinding: not clear. Incomplete outcome data: no. Stopped early for benefit/arm: no.
	Participants	1180 postmenopausal women aged at least 55 yr. Calcium plus VitD: 446 women; Breast cancers: 5/446. Calcium: 445 women; Breast cancers: 6/445.
	Interventions	Participants were randomly assigned to receive 1400–1500 mg supplemental calcium/vit D alone, supplemental calcium plus 1100 UI vit D or placebo for 4 yr.
	Outcomes	Cancer incidence.

The first study was a randomized factorial-design trial of cholecalciferol and calcium supplementation for the secondary prevention of fragility fractures in 5292 people aged 70 years and older. Women represented about 85% of the overall sample. Participants were randomized to daily cholecalciferol (800 IU), calcium (1000 mg), both, or placebo for 24–62 months, with a minimum follow up for long term outcomes of three years. Cancer incidence and mortality were listed among the secondary outcomes along with overall mortality and mortality from vascular diseases. Overall, the study did not rule out a significant role of vit D or calcium on cancer incidence and mortality (HR: 1.06, 95% CI: 0.91–1.24, and HR: 1.03, 95% CI: 0.94–1.13, respectively) [30].

Lappe and co-authors carried out a 4-year, placebo-controlled, randomized trial to assess the impact of calcium and cholecalciferol supplementation on the skeletal status and calcium homeostasis in healthy postmenopausal women aged 55 years and older. The 1180 participants were randomized to placebo, consisting of both a vit D placebo and calcium placebo, calcium (1400 mg/day of calcium citrate or 1500 mg/day of calcium carbonate) and a vit D placebo, calcium and 1000 IU cholecalciferol/day. Cancer incidence was a secondary outcomes. The study results showed a decreased risk of cancer development in women randomized to calcium and cholecalciferol as well as in participants taking calcium compared with placebo (RR: 0.402, p = 0.001 and RR: 0.532, p = 0.06, respectively) [21].

### Risk of Bias in Included Studies

**Figure 2:**
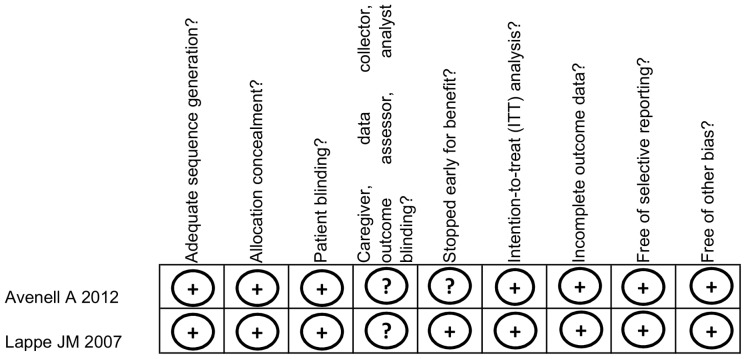
Risk of bias summary. Legend to Figure 2. Reviewers’ judgment on each “Risk of bias” item within each included study.

Overall results of risk of bias assessment appear in figure 2.

In both RCTs, randomization was centralized, computer-generated. Participating women were blind to treatment assignment due the use of placebo. No specific details are reported on whether blinding was extended to caregivers, data collectors, outcome assessors and analysts.

In the study from Lappe and co-authors, about 13.2% of the study participants (156 out of 1180) were lost to follow up. Reasons for drop out and droppers’ distribution across the study arms are not reported. Incomplete outcome data are not addressed by Avenell et al.

We found no evidence of selective reporting for the two studies included. In both the RCTs, the outcomes listed in the methods section of the cited manuscripts fully matched with those reported by the original protocols [119]–[120].

None of the two RCTs was stopped earlier. Authors from both RCTs reported conducting ITT analyses. However, in Lappe and co-authors the number of participants randomized to treatment assignment does not exactly match with the number of participants followed up due to the exclusion of one woman after randomization. Reasons for exclusion are described in details. We could not identify further potential sources of bias for the studies included.

### Effects of Intervention

#### Breast cancer incidence

Both the included trials contributed patients to this outcome.

All the 4481 women participating in the trial from Avenell were included. By pre-set inclusion criteria calling for comparison of study groups differing by vit D use only, participants allocated to cholecalciferol and calcium were compared to participants taking calcium (Avenell A 2012-1^st^), while women in the vit D arm were compared to participants randomized to placebo (Avenell A 2012-2^nd^). Conversely, the study from Lappe et al contributed only partly to the quantitative synthesis on breast cancer incidence. Indeed, we included participants randomized to cholecalciferol and calcium (446/1180) and compared them to women allocated to calcium only (445/1180). Study participants assigned to placebo were not considered (288/1180). Women participating in this trial tended to be significantly younger and with higher serum concentration of 25-OH vit D at baseline (i.e. 71±20.3 nmol/liter) compared to those participating in the trial from Avenell and colleagues (Table 1). The most significant risk reduction was observed in women randomized to vit D and calcium compared with placebo (0.402, p = 0.01). This was the only subgroup with a significant change in circulating levels of 25-OH vit D at 12 months compared to baseline concentrations (i.e. absolute change 23.9±17.8).

**Figure 3:**
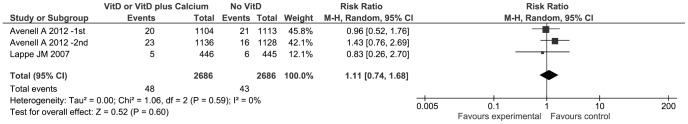
Forest plot of vitamin D supplementation and breast cancer incidence. Legend to Figure 3. M–H, Mantel-Haenszel; 95% CI, 95% confidence interval; df, degrees of freedom.

**Figure 4:**
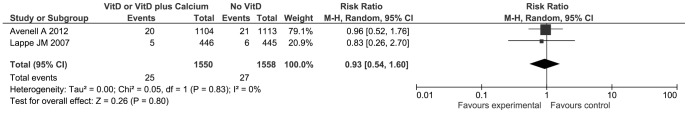
Forest plot of vitamin D supplementation and breast cancer incidence. Administration mode on treatment effects. Legend to Figure 4. M–H, Mantel-Haenszel; 95% CI, 95% confidence interval; df, degrees of freedom.

The pooled analysis included 5372 women. Overall, RRs and 95% CI were 1.11 and 0.74–1.68, respectively. We found no evidence of significant heterogeneity (p = 0.59, I^2^: 0%) (figure 3). Meta-regression analysis of vit D dosage performed on the basis of a random effects model methodology showed non-significant results. However, there was a suggestion for greater treatment efficacy with higher vit D dosage (RR 0.58, 95% CI 0.23–1.47). In analysis of mode of administration, women taking vit D and calcium appeared somewhat less likely to develop breast cancer compared with subjects receiving calcium and placebo, though at a non significant extent (0.93, 0.54–1.59; I^2^: 0%) (figure 4).

#### Breast cancer mortality

The number of breast cancer-related fatal events was available for one single study at three years of follow up. Deaths from breast cancer out of the total events due to cancer by study arm were as it follows: 7/78, 7/73, 9/95 and 4/83 in women allocated to vit D and calcium, vit D and (calcium) placebo, (vit D) placebo and calcium or placebo, respectively. The lack of point-in-time data refrained us from extracting the log HR and its variance for breast cancer mortality. However, since mortality data were provided exclusively by one trial [30], the meta-analytical approach was substantially not feasible.

## Discussion

We conducted a systematic review of RCTs focused on vit D supplemenation in breast cancer prevention. Based on pre-stated selection criteria, only two trials were included. Results from the pooled analysis do not support a role of vit D in reducing breast cancer risk in postmenopausal women. When compared to controls allocated to calcium and/or placebo, healthy women taking vitamin D either as a single agent or part of a combined treatment did not show a reduced risk of breast cancer development. In sensitivity analysis, vit D dosage and mode of administration did not affect the outcome of interest at a significant extent, although treatment efficacy appeared somewhat greater when vit D was administered at the highest dosage and in combination with calcium.

This systematic review has the following strengths. We followed the Cochrane Collaboration methods for the conduct of systematic reviews of intervention trials. A qualified librarian designed an extensive search strategy, which was applied with sensitivity criteria to the first phase of the reference screening. The search of three major databases, use of the related tools and other reference sources, along with the lack of language restrictions, increase our confidence in the identification of all relevant trials.

There are some limitations to this review. Our efforts to identify published trials were not paralleled by the systematic attempt to locate unpublished studies. Moreover, we could not assess publication bias by funnel plots due to the restricted number of RCTs included. On this basis, publication bias cannot be excluded. Among unpublished trials, those with non-significant results represent the vast majority [121]. It is plausible to hypothesize that, if existing, unpublished studies on the effects of vit D in breast cancer prevention would not have added significant evidence in support of a beneficial effect of the intervention of interest, thus eventually strengthening our conclusions.

In addition, all women participating in the included studies were postmenopausal. This limits our ability to make inference regarding vit D use for breast cancer prevention in healthy premenopausal women. The number of studies judged eligible was particularly low. This result, along with the relatively low number of women included (i.e. 5372) and the paucity of breast cancer events (i.e. 48 and 43 breast cancers in women allocated to the intervention and control group, respectively), might have conferred to our meta-analysis an insufficient power to detect the effects of interest. Moreover, when singularly considered, none of the included studies was primarily tailored to detect incident cancers, either overall or by primitive cancer site. Our results were largely driven by the RCT from Avenell and co-authors. Women participating in this trial were older and had poorer vit D status at recruitment than those participating in the other trial. However, baseline, circulating levels of 25-OH vit D were assessed in only a very small subgroup of 60 participants and might not be representative of the wider trial population. In this same group, supplementation was associated with an increase in circulating 25-OH vit D from 38 to 62 nmol/liter, which could still be suboptimal [122].

Dose adequacy represents a further, critical issue. Adults aged 50–70 and 70 years and older require at least 600 and 800 IU of vit D daily, respectively. However, to raise the blood level of 25-OH vit D above 75 nmol/liter requires at least 1500–2000 IU daily [123]. In the trial from Avenell, participants received 800 IU of vit D for 24–62 months, with a minimum follow up for long term outcomes of three years. Lappe and co-authors set up the daily supplementation of vit D at 1100 IU. Only in this latter study, the circulating 25-OH vit D levels raised to recommended values. Furthermore, the duration of the included studies could be questionable for long-term outcomes. The 4- and 5-year duration of the trials from Lappe and Avenell might be insufficient to the detection of incident cancer cases.

When combined with calcium, vit D showed a slight, not significant protective role towards breast cancer development compared with calcium alone (RR 0.93, 95% CI: 0.54–1.59, I^2^ 0%). This could suggest that the reduced risk of cancer development is due to the effect of calcium rather than vit D. However, the inclusion of RCTs with co-interventions balanced across the compared groups should have minimized the differential contribution of calcium to the overall treatment effects.

To our knowledge, this is the first systematic review specifically conceived to provide the reader with a comprehensive appraisal of RCTs focused on vit D supplementation in breast cancer prevention. Breast cancer-related outcomes were not included in the systematic review from Autier and Gandini, neither did ReJnmark report on cancer mortality [18], [24]. Conversely, two trials on vit D in breast cancer prevention were considered eligible by Chung and co-authors [21]–[22]. Our systematic review does not include the trial carried out by the Women’s Health Initiative Investigators because of unbalanced co-interventions between the study arms. In this study, postmenopausal women were randomized to 1000 milligrams of elemental calcium and 400 IU of daily cholecalciferol or placebo. This study findings do not support a role of vit D supplementation in reducing breast cancer incidence (HR: 0.96, 95% CI: 0.85–0.96) [22]. Critics have been fueled by the low vit D dose along with poor treatment adherence and off study use of additional vit D and calcium supplements [23], [124]–[125]. The inclusion of the trial from Avenell represents a further distinctive feature of our work, since results from this trial were not available at the time of the previous systematic reviews’ conduct and publication [30].

In summary, our work contributes a systematic appraisal of the currently available randomized controlled evidence on vit D supplementation for breast cancer prevention. Based on our results and limited trial evidence, vit D supplementation seems not to be associated with a reduced risk of breast cancer development in postmenopausal women. However, the scientific panorama related to the association of interest is still limited and inadequate to draw firm conclusions. The lack of systematic assessment of vit D status applied to the entire study population in the widest trial included, along with the documented potentialities for dose inadequacy and insufficient study length relatively to cancer-related outcomes, represent major limitations of the literature examined. New trials specifically tailored on the vit D-cancer- binomious are in progress and should provide additional information in a few years’ time [126]–[127]. Methodological tools and key tenets of vit D metabolism and biological activities will help interpret the upcoming results.

## Supporting Information

Appendix S1 **Search strategy.**
(DOCX)
