# Supplementary material for: Vitamin D Supplementation and Breast Cancer Prevention: A Systematic Review and Meta-Analysis of Randomized Clinical Trials
Source: PLoS One. 2013 Jul 22;8(7):e69269. doi: 10.1371/journal.pone.0069269 (PMC3718745; doi:10.1371/journal.pone.0069269)
Supplement: Appendix S1 — Search strategy. (DOCX) [file pone.0069269.s001.docx]

**APPENDIX S1**

Database: Central

Search Strategy:

--------------------------------------------------------------------------------

#1 (malignan$ or neoplasm$ or cancer or carcinoma$ or adenocarcinoma or tumour or tumor or sarcoma$ or lymphedema or dcis) or (ductal or infiltrating or intraductal or lobular or medullary) or (breast or mammary) in Clinical Trials

#2 (vit D or cholecalciferol or colecalciferol or hydroxycholecalciferol or hydroxycolecalciferol or dihydroxycholecalciferol or dihydroxycolecalciferol or dihydrotachysterol or maxicalcitol or oxacalcitriol or paricalcitol or doxercalciferol or dihydroxyvitamin or falecalcitriol or calcitriol or alfacalcidol or alphacalcidol or calcifedol or calcipotriol or epicalcitriol or lexacalcitol or seocalcitol or tacalcitol or ergocalciferol or Hydroxyvit D) in Clinical Trials #3 (#1 AND #2)

Database: EMBASE

Search Strategy:

--------------------------------------------------------------------------------

1 (vit D or cholecalciferol or colecalciferol or hydroxycholecalciferol or hydroxycolecalciferol or dihydroxycholecalciferol or dihydroxycolecalciferol or dihydrotachysterol or maxicalcitol or oxacalcitriol or paricalcitol or doxercalciferol or dihydroxyvitamin or falecalcitriol or calcitriol or alfacalcidol or alphacalcidol or calcifedol or calcipotriol or epicalcitriol or lexacalcitol or seocalcitol or tacalcitol or ergocalciferol or Hydroxyvit D).tw.

2 exp vit D/

3 1 or 2

4 exp neoplasm/

5 (malignan$ or neoplasm$ or cancer or carcinoma$ or adenocarcinoma or tumour or tumor or sarcoma$ or lymphedema or dcis).tw.

6 (ductal or infiltrating or intraductal or lobular or medullary).tw.

7 (breast or mammary).tw.

8 or/4-7

9 random*.tw.

10 clinical trial*.mp.

11 exp health care quality/

12 or/9-11

13 3 and 8 and 12

Database: Ovid MEDLINE(R) In-Process & Other Non-Indexed Citations and Ovid MEDLINE(R)

Search Strategy:

--------------------------------------------------------------------------------

1 randomized controlled trial.pt.

2 controlled clinical trial.pt.

3 randomized.ab.

4 placebo.ab.

5 drug therapy.fs.

6 randomly.ab.

7 trial.ab.

8 groups.ab.

9 1 or 2 or 3 or 4 or 5 or 6 or 7 or 8

10 (animals not (humans and animals)).sh.

11 9 not 10

12 vit D/

13 24,25-dihydroxyvit D 3/

14 25-hydroxyvit D 2/

15 calcifediol/

16 calcitriol/

17 cholecalciferol/

18 dihydroxycholecalciferols/

19 ergocalciferols/

20 hydroxycholecalciferols/

21 or/12-20

22 (vit D or cholecalciferol or colecalciferol or hydroxycholecalciferol or hydroxycolecalciferol or dihydroxycholecalciferol or dihydroxycolecalciferol or dihydrotachysterol or maxicalcitol or oxacalcitriol or paricalcitol or doxercalciferol or dihydroxyvitamin or falecalcitriol or calcitriol or alfacalcidol or alphacalcidol or calcifedol or calcipotriol or epicalcitriol or lexacalcitol or seocalcitol or tacalcitol or ergocalciferol or Hydroxyvit D).tw.

23 21 or 22

24 neoplasms/

25 (malignan$ or neoplasm$ or cancer or carcinoma$ or adenocarcinoma or tumour or tumor or sarcoma$ or lymphedema or dcis).tw.

26 (ductal or infiltrating or intraductal or lobular or medullary).tw.

27 (breast or mammary).tw.

28 or/24-27

1. 23 and 28 and 11
